# Supplementary material for: Analysis Methods for Diagnosing Rare Neurodevelopmental Diseases with Episignatures: A Systematic Review of the Literature
Source: Biomedicines. 2025 Dec 11;13(12):3043. doi: 10.3390/biomedicines13123043 (PMC12730964; doi:10.3390/biomedicines13123043)
Supplement: Supplementary file 1 [file biomedicines-13-03043-s001.zip › File S2-Supplementary methods.pdf]

SUPPLEMENTARY MATERIAL: Systematic Review Filters

Systematic review methodological structure.

- Reviewer: A.A.-G.,
- Article identification: A.A.-G., A.C., M.S.-P., L.A.P.-J., J.R.G.
- Each article was reviewed manually, no automated tools were used in any step.

(1) PUBMED search (2024/12):

- Query: Episignature* OR Epimutation*.
- Time filters: 2014 – 2024.
- Keywords present in title, abstract, keywords or full text.

Step 1: Test accessibility (n = 667)	Text available in English, and accessible by our institution (CERCA consortium or UAB).<br>(n = 2 → Not accessible)
Step 2: article screening by title and keywords.<br>(n = 665)	Inspect if the article was a novel episignature or detected epimutations in Rare Diseases.<br>Title/Keywords filter:<br>- Signature.<br>- Epigenetic.<br>- Profiling/Screening.<br>- DNA methylation.<br>(n = 571 → did not met this criteria).

94 articles passed both filters.

(2) PubMed weekly review throughout 2025 by the team, returned 7 articles.

- o Discovery of a DNA methylation profile in individuals with Sifrim-Hitz-Weiss syndrome.
- o ARID2-related disorder: further delineation of the clinical phenotype of 27 novel individuals and description of an epigenetic signature.
- o Pleiotropic effects of MORC2 derive from its epigenetic signature.
- o Dominant variants in major spliceosome U4 and U5 small nuclear RNA genes cause neurodevelopmental disorders through splicing disruption.
- o Discovery of a DNA methylation episignature as a molecular biomarker for fetal alcohol syndrome.
- o PTBP1 variants displaying altered nucleocytoplasmic distribution are responsible for a neurodevelopmental disorder with skeletal dysplasia.
- o Characterization of snRNA-related neurodevelopmental disorders through the Spanish Undiagnosed Rare Disease Programs.

(3) Article identification by references. List of source articles:

- Reviews/multi-disease (n = 32):
  - o EpigenCentral: Portal for DNA Methylation Data Analysis and Classification in Rare Diseases.
  - o Evaluation of DNA Methylation Episignatures for Diagnosis and Phenotype Correlations in 42 Mendelian Neurodevelopmental Disorders.

- Training with Synthetic Data Provides Accurate and Openly-Available DNA Methylation Classifiers for Developmental Disorders and Congenital Anomalies via MethaDory.
- A Survey of Rare Epigenetic Variation in 23,116 Human Genomes Identifies Disease-Relevant Epivariations and CGG Expansions

- Studies from the search (n = 27).
    - Epigenetics of Autism Spectrum Disorders: A Multi-level Analysis Combining Epi-signature, Age Acceleration, Epigenetic Drift and Rare Epivariations Using Public Datasets
    - Detection of a DNA Methylation Signature for the Intellectual Developmental Disorder, X-Linked, Syndromic, Armfield Type.
    - PRDX1 gene-related epi-cblC disease is a common type of inborn error of cobalamin metabolism with mono- or bi-allelic MMACHC epimutations.
    - Genome-wide methylation analysis in Silver-Russell syndrome patients.
    - An Epigenetic Biomarker for Adult High-Functioning Autism Spectrum Disorder.

All articles were redirected to A.A.-G. for step 3 inspection.

Step 3: article screening by abstract + methods. (n = 160)	Inspect if articles not complied with the following:<br>- Blood assays (n = 8).<br>- Episignature development (n = 24).<br>- Case/Control comparisons (n = 9).<br>- No case-reports (n = 11).

List of excluded articles in Step 3. Some excluded articles could fit in more than one category, we indicated the ones we considered fitted best. Articles included are indicated in the main text.

No Blood assays	- DNA methylation as a predictor of fetal alcohol spectrum disorder<br>- DNA methylation signature of human fetal alcohol spectrum disorder<br>- Genome-wide DNA methylation analysis in multiple tissues in primary Sjögren's syndrome reveals regulatory effects at interferon-induced genes<br>- In skeletal muscle and neural crest cells, SMCHD1 regulates biological pathways relevant for Bosma syndrome and facioscapulohumeral dystrophy phenotype.<br>- Epimutations are associated with CHROMOMETHYLASE 3-induced de novo DNA methylation.<br>- Epigenetic Delay in the Neurodevelopmental Trajectory of DNA Methylation States in Autism Spectrum Disorders.<br>- High-resolution, noninvasive single-cell lineage tracing in mice and humans based on DNA methylation epimutations.<br>- Global DNA methylation differences involving germline structural variation impact gene expression in pediatric brain tumors
No developed a new episignature or	- DNA methylation profiling in Kabuki syndrome: reclassification of germline KMT2D VUS and sensitivity in validating postzygotic mosaicism.

studied new epimutations	- Episignatures in practice: independent evaluation of published episignatures for the molecular diagnostics of ten neurodevelopmental disorders<br>- A Multi-Omic Approach Identifies an Autism Spectrum Disorder (ASD) Regulatory Complex of Functional Epimutations in Placentas from Children Born Preterm; Epigenome-wide DNA methylation in placentas from preterm infants: association with maternal socioeconomic status<br>- DNA methylation signature classification of rare disorders using publicly available methylation data.<br>- Rare Variants in 48 Genes Account for 42% of Cases of Epilepsy With or Without Neurodevelopmental Delay in 246 Pediatric Patients.<br>- Clinical epigenomics: genome-wide DNA methylation analysis for the diagnosis of Mendelian disorders.<br>- Functional annotation of genomic variation: DNA methylation episignatures in neurodevelopmental Mendelian disorders.<br>- Molecular signatures in Mendelian neurodevelopment: a focus on ubiquitination driven DNA methylation aberrations.<br>- Molecular characterisation of 36 multilocus imprinting disturbance (MLID) patients: a comprehensive approach.<br>- Congenital hyperinsulinism and novel KDM6A duplications - resolving pathogenicity with genome and epigenetic analyses.<br>- Genome-wide DNA methylation profiling confirms a case of low-level mosaic Kabuki syndrome 1.<br>- Epimutation of MMACHC compound to a genetic mutation in cblC cases.<br>- Kagami-Ogata syndrome: a clinically recognizable upd(14)pat and related disorder affecting the chromosome 14q32.2 imprinted region.<br>- Prenatal molecular testing for Beckwith-Wiedemann and Silver-Russell syndromes: a challenge for molecular analysis and genetic counselling.<br>- Methylation assay in KMT2B-related dystonia: a novel diagnostic validation tool.<br>- Molecular characterization of temple syndrome families with 14q32 epimutations.<br>- Chromosome 14q32.2 Imprinted Region Disruption as an Alternative Molecular Diagnosis of Silver-Russell Syndrome.<br>- Comprehensive clinical studies in 34 patients with molecularly defined UPD(14)pat and related conditions (Kagami-Ogata syndrome).<br>- DNA Methylation and Susceptibility to Autism Spectrum Disorder.<br>- Concomitant mutation and epimutation of the MLH1 gene in a Lynch syndrome family.<br>- Novel deletions affecting the MEG3-DMR provide further evidence for a hierarchical regulation of imprinting in 14q32.<br>- Finding the needle in a haystack: identification of cases of Lynch syndrome with MLH1 epimutation.<br>- Decreased expression of cell proliferation-related genes in clonally derived skin fibroblasts from children with Silver-Russell

	- syndrome is independent of the degree of 11p15 ICR1 hypomethylation.<br>- A PRDX1 mutant allele causes a MMACHC secondary epimutation in cblC patients
No Case/Control approach	- A Survey of Rare Epigenetic Variation in 23,116 Human Genomes Identifies Disease-Relevant Epivariations and CGG Expansions.<br>- A Comprehensive Approach to the Diagnosis of Leigh Syndrome Spectrum<br>- A roadmap to cure CHD2-related disorders.<br>- DNA methylation changes in the genome of patients with hypogonadotropic hypogonadism.<br>- Examinations of maternal uniparental disomy and epimutations for chromosomes 6, 14, 16 and 20 in Silver-Russell syndrome-like phenotypes.<br>- A New EP300 -Related Syndrome With Prominent Developmental and Immune Phenotypes<br>- Intellectual functioning in Silver-Russell syndrome: First study in adults.<br>- Case-control meta-analysis of blood DNA methylation and autism spectrum disorder.<br>- Diagnostic utility and reporting recommendations for clinical DNA methylation episignature testing in genetically undiagnosed rare diseases
A case-report.	- Genetically unresolved case of Rauch-Steindl syndrome diagnosed by its wolf-hirschhorn associated DNA methylation episignature.<br>- Identification of a KDM6A somatic mutation responsible for Kabuki syndrome by excluding a conflicting KMT2D germline variant through episignature analysis.<br>- DNA methylation analysis in patients with neurodevelopmental disorders improves variant interpretation and reveals complexity.<br>- Integration of EpiSign, facial phenotyping, and likelihood ratio interpretation of clinical abnormalities in the re-classification of an ARID1B missense variant.<br>- Missense variant in SRCAP with distinct DNA methylation signature associated with non-FLHS SRCAP-related neurodevelopmental disorder.<br>- Diagnosis of TET3-Related Beck-Fahrner Syndrome in an Individual With Chorioretinal and Iris Colobomata Using a DNA Methylation Signature<br>- A case of an Angelman-syndrome caused by an intragenic duplication of UBE3A uncovered by adaptive nanopore sequencing.<br>- Contribution of DNA methylation profiling to the reclassification of a variant of uncertain significance in the KDM5C gene.<br>- The phenotypic spectrum and genotype-phenotype correlations in 106 patients with variants in major autism gene CHD8.<br>- What Have We Learned from Patients Who Have Arboleda-Tham Syndrome Due to a De Novo KAT6A Pathogenic Variant with Impaired Histone Acetyltransferase Function? A Precise Clinical

	Description May Be Critical for Genetic Testing Approach and Final Diagnosis.<br>- Beckwith-Wiedemann syndrome with long QT caused by a deletion involving KCNQ1 but not KCNQ1OT1:TSS-DMR.
