# Supplementary material for: Analysis Methods for Diagnosing Rare Neurodevelopmental Diseases with Episignatures: A Systematic Review of the Literature
Source: Biomedicines. 2025 Dec 11;13(12):3043. doi: 10.3390/biomedicines13123043 (PMC12730964; doi:10.3390/biomedicines13123043)
Supplement: Supplementary file 1 [file biomedicines-13-03043-s001.zip › Supplementary-SuppTables.pdf]

Table S1: syndromes included in EpiSign (version 5). Adapted from: *Methylation Array Panel content for EpiSign version 5. NWGLH in partnership with EpiSign, London Health Science Centre*.

Disorder	Gene or Region	Notes
Cerebellar ataxia, deafness, and narcolepsy, autosomal dominant	*DNMT1*	Reduced sensitivity may be observed.
ARID1A duplication-related syndrome	*ARID1A*	The range of validated coordinates is 1p36.11(26,964,202- 27,099,490). CNVs overlapping or expanding this region may also be detected. Reduced sensitivity may be observed.
Arboleda-Tham syndrome	*KAT6A*	Reduced sensitivity may be observed.
Alpha-thalassemia/Impaired intellectual development syndrome, X-linked	*ATRX*	Episignature defined with male cases only. Heterozygotes have been shown to not match the episignature.
BAFopathies: Coffin-Siris (CSS1-4) & Nicolaides-Baraitser (NCBRS) syndromes	*ARID1A, ARID1B, SMARCB1, SMARCA4, SMARCA2*	Patients with other BAFopathy genes may be detected.
Branchial arch abnormalities, choanal atresia, athelia, hearing loss, and hypothyroidism syndrome	*KMT2D*	Only for variants within the amino acid range of 3400-3700. Reduced sensitivity may be observed.
Beck-Fahrner syndrome	*TET3*	Healthy carriers and those with incomplete penetrance are detectable. Patients with biallelic variants are distinguishable from those with monoallelic variants.
Börjeson-Forssman-Lehmann syndrome	*PHF6*	This is a secondary episignature; sample must also be positive for the combined Börjeson-Forssman-Lehmann, Chung-Jansen and White Kernohan syndromes signature. Episignature defined with male cases only. Heterozygotes have been shown to not match the episignature. Reduced sensitivity may be observed.
Blepharophimosis-impaired intellectual development syndrome	*SMARCA2*	
Cornelia de Lange syndromes 1-4	*NIPBL, RAD21, SMC3, SMC1A*	
Cornelia de Lange syndrome 1	*NIPBL*	This is a secondary episignature; sample must also be positive for Cornelia de Lange syndromes 1-4 signature. Reduced sensitivity may be observed.
Cornelia de Lange syndrome 2	*SMC1A*	This is a secondary episignature; sample must also be positive for Cornelia de Lange syndromes 1-4 signature. Reduced sensitivity may be observed.
Cornelia de Lange syndrome 3	*SMC3*	This is a secondary episignature; sample must also be positive for Cornelia de Lange syndromes 1-4 signature. Reduced sensitivity may be observed.

Cornelia de Lange syndrome 4	*RAD21*	This is a secondary episignature; sample must also be positive for Cornelia de Lange syndromes 1-4 signature. Reduced sensitivity may be observed.
CHARGE syndrome	*CHD7*	
Congenital heart defects, dysmorphic facial features, and intellectual developmental disorder	*CDK13, CCNK*	Reduced sensitivity may be observed.
Chromosome 19p13.13 deletion syndrome	Chr19p13.13p13.2 deletion	The range of validated coordinates is 19p13.13p13.2(13,201,983-13,213,144). CNVs overlapping or expanding this region may also be detected. Only for copy number variants. NFIX sequence variants have been shown to not match the episignature.
Chromosome 1p36 deletion syndrome	Chr1p36 deletion	The range of validated coordinates is 1p36.33p36.32(1,019,753- 2,867,961). CNVs overlapping or expanding this region may also be detected. Reduced sensitivity may be observed.
Chromosome Xp11.22 duplication syndrome	ChrXp11.22 duplication	The range of validated coordinates is Xp11.22(53,559,057- 53,654,518). CNVs overlapping or expanding this region may also be detected. Episignature defined with male cases only. Heterozygotes have been shown to not match the episignature. Reduced sensitivity may be observed.
Börjeson-Forssman-Lehmann, Chung-Jansen and White Kernohan syndromes	*PHIP, PHF6, DDB1*	
Chung-Jansen syndrome	*PHIP*	This is a secondary episignature; sample must also be positive for the combined Börjeson-Forssman-Lehmann, Chung-Jansen and White Kernohan syndromes signature. Reduced sensitivity may be observed.
Clark-Baraitser syndrome	*TRIP12*	
BAFopathies: Coffin-Siris syndrome 1 & 2	*ARID1A, ARID1B*	Only for variants near c.6200.
Coffin-Siris syndrome 1	*ARID1B*	This is a secondary episignature; sample must also be positive for BAFopathy. Reduced sensitivity may be observed.
Coffin-Siris syndrome 2	*ARID1A*	This is a secondary episignature; sample must also be positive for BAFopathy. Reduced sensitivity may be observed.
Coffin-Siris syndrome 3	*SMARCB1*	This is a secondary episignature; sample must also be positive for BAFopathy. Reduced sensitivity may be observed.
Coffin-Siris syndrome 4	*SMARCA4*	This is a secondary episignature; sample must also be positive for BAFopathy. Reduced sensitivity may be observed.
Coffin-Siris syndrome 4	*SMARCA4*	Only for variants near c.2656. No separate episignature due small cohort size however these samples cluster separately from other BAFopathy/CSS4 samples.

Coffin-Siris syndrome 6	*ARID2*	
Developmental and epileptic encephalopathy 54	*HNRNPU*	
Developmental and epileptic encephalopathy 94	*CHD2*	
DEGCAGS syndrome	*ZNF699*	Heterozygotes have been shown to not match the episignature.
Developmental delay with variable intellectual disability and dysmorphic facies	*JARID2*	Reduced sensitivity may be observed.
Diets-Jongmans syndrome	*KDM3B*	
Down syndrome	Chr21 trisomy	
Williams-Beuren region duplication syndrome	Chr7q11.23 duplication	The range of validated coordinates is 7q11.23(73,953,518- 74,138,459). CNVs overlapping or expanding this region may also be detected.
Dystonia 28, childhood-onset	*KMT2B*	
Fanconi anemia	*FANCA, FANCC, FANCD2, FANCG, FANCI, FANCL*	Heterozygotes have been shown to not match the episignature. Patients with other FANC genes may be detected.
Floating Harbour syndrome	*SRCAP*	
Gabriele-de Vries syndrome	*YY1*	Reduced sensitivity may be observed.
Genitopatellar syndrome	*KAT6B*	Reduced sensitivity may be observed. Since GTPTS and SBBYSS are both caused by variants in KAT6B, it is recommended to request both episignatures for VUS assessment.
Hao-Fountain syndrome	*USP7*	
Hunter McAlpine craniosynostosis syndrome	Chr5q35 duplication involving NSD1	The range of validated coordinates is 5q35.2q35.3(175,839,681- 176,904,798). CNVs overlapping or expanding this region may also be detected.
Helsmoortel-van der Aa syndrome	*ADNP*	Central episignature for variants within the coding nucleotide range of c.2054-2340.
Helsmoortel-van der Aa syndrome	*ADNP*	Terminal episignature for variants outside of the coding nucleotide range of c.2054-2340.
Immunodeficiency-centromeric instability-facial anomalies syndrome 1	*DNMT3B*	Reduced sensitivity may be observed.
Immunodeficiency-centromeric instability-facial anomalies syndrome 2-4	*CDCA7, ZBTB24, HELLS*	Reduced sensitivity may be observed.
Intellectual developmental disorder with autism and macrocephaly	*CHD8*	Reduced sensitivity may be observed.
Intellectual developmental disorder with microcephaly and with or without ocular malformations or hypogonadotropic hypogonadism	*SOX11*	Reduced sensitivity may be observed.

Intellectual developmental disorder with seizures and language delay	*SETD1B*	
Intellectual developmental disorder with dysmorphic facies, speech delay, and T-cell abnormalities	*BCL11B*	Reduced sensitivity may be observed.
Kabuki syndrome 1 & 2	*KMT2D, KDM6A*	
Kabuki syndrome 1	*KMT2D*	This is a secondary episignature; sample must also be positive for Kabuki. Reduced sensitivity may be observed.
Kabuki syndrome 2	*KDM6A*	This is a secondary episignature; sample must also be positive for Kabuki. Reduced sensitivity may be observed.
KBG syndrome	*ANKRD11*	This is a secondary episignature; sample must also be positive for KBGS_MRD23. Reduced sensitivity may be observed.
Intellectual developmental disorder, autosomal dominant 23; KBGS syndrome	*SETD5, ANKRD11*	
KDM2B-related syndrome	*KDM2B*	
Koolen de Vreis syndrome	*KANSL1*	
Kleefstra syndrome 1	*EHMT1*	
Luscan-Lumish syndrome	*SETD2*	
Menke-Hennekam syndrome 1 & 2	*CREBBP, EP300*	Only for domain ID4. MKHK1 and MKHK2 exhibit a shared ID4 domain episignature and therefore cannot distinguish between MKHK1 and MKHK2. Other domains of MKHK1/2 are not available.
Mowat-Wilson syndrome	*ZEB2*	
Intellectual developmental disorder, autosomal dominant 21	*CTCF*	
Intellectual developmental disorder, autosomal dominant 23	*SETD5*	This is a secondary episignature; sample must also be positive for KBGS_MRD23. Reduced sensitivity may be observed.
Intellectual developmental disorder, autosomal dominant 51	*KMT5B*	Healthy carriers and those with incomplete penetrance are detectable. Reduced sensitivity may be observed.
Intellectual developmental disorder, autosomal dominant 7	*DYRK1A*	
Intellectual developmental disorder, X-linked, syndromic, Armfield type	*FAM50A*	Episignature defined with male cases only. Heterozygotes have been shown to not match the episignature. Reduced sensitivity may be observed.
Intellectual developmental disorder, X-linked, syndromic, Claes-Jensen type	*KDM5C*	Healthy carriers and those with incomplete penetrance are detectable. Heterozygotes have a distinct profile from hemizygotes.
Intellectual developmental disorder, X-linked syndromic, Nascimento type	*UBE2A*	Episignature defined with male cases only. Heterozygotes have been shown to not match the episignature. Reduced sensitivity may be observed.
Intellectual developmental disorder, X-linked, syndromic, Snyder-Robinson type	*SMS*	Episignature defined with male cases only. Reduced sensitivity may be observed.

MSL2-related syndrome	*MSL2*	Reduced sensitivity may be observed.
Nicolaides-Baraitser syndrome	*SMARCA2*	This is a secondary episignature; sample must also be positive for BAFopathy. Reduced sensitivity may be observed.
Neurodevelopmental disorder with dysmorphic facies and behavioral abnormalities	*SRSF1*	
Neurodevelopmental disorder with hypotonia, stereotypic hand movements, and impaired language	*MEF2C*	
NSD2 duplication-related syndrome	*NSD2*	The range of validated coordinates is 4p16.3(1,832,733- 1,975,031). CNVs overlapping or expanding this region may also be detected.
Phelan-McDermid syndrome	Chr22q13.3 deletion	The range of validated coordinates is 22q13.3(49,238,268- 50,248,907). CNVs overlapping or expanding this region may also be detected. Only for copy number variants. SHANK3 sequence variants have been shown to not match the episignature.
PRC2 complex disorders (Weaver and Cohen-Gibson syndromes)	*EZH2, EED*	Shared episignature between PRC2 complex syndromes WVS and COGIS. IMMAS (Imagawa-Matsumoto syndrome) cases with variants in SUZ12 have also been detected.
Neuroocular syndrome	*PRR12*	Healthy carriers and those with incomplete penetrance are detectable. Reduced sensitivity may be observed.
Pitt-Hopkins syndrome	*TCF4*	
Potocki-Lupski syndrome	Chr17p11.2 duplication	The range of validated coordinates is 17p11.2(16,779,412- 20,231,379). CNVs overlapping or expanding this region may also be detected. Reduced sensitivity may be observed.
Renpenning syndrome	*PQBP1*	Episignature defined with male cases only. Heterozygotes have been shown to not match the episignature. Reduced sensitivity may be observed.
Rahman syndrome	*H1-4*	
Rubinstein-Taybi syndrome 1 and 2	*CREBBP, EP300*	
Rubinstein-Taybi syndrome 1	*CREBBP*	This is a secondary episignature; sample must also be positive for RSTS.
Rubinstein-Taybi syndrome 2	*EP300*	This is a secondary episignature; sample must also be positive for RSTS.
Ohdo syndrome, SBBYSS variant	*KAT6B*	Reduced sensitivity may be observed. Since GTPTS and SBBYSS are both caused by variants in KAT6B, it is recommended to request both episignatures for VUS assessment.
Sifrim-Hitz-Weiss syndrome	*CHD4*	
SLC32A1-related syndrome	*SLC32A1*	Reduced sensitivity may be observed.
Smith-Magenis syndrome	Chr17p11.2 deletion	The range of validated coordinates is 17p11.2(17,322,913- 18,515,769). CNVs overlapping or expanding this region may also

		be detected. Only for copy number variants. RAI1 sequence variants have been shown to not match the episignature.
Sotos syndrome	*NSD1*	
Tatton-Brown-Rahman syndrome	*DNMT3A*	Reduced sensitivity may be observed.
Turner syndrome	ChrX deletion; 45,X	
Velocardiofacial syndrome	Chr22q11.2 deletion	The range of validated coordinates is 22q11.21(19,510,547- 20,285,090). CNVs overlapping or expanding these regions may be detected.
Wiedemann-Steiner syndrome	*KMT2A*	
White-Kernohan syndrome	*DDB1*	This is a secondary episignature; sample must also be positive for the Börjeson-Forssman-Lehmann, Chung-Jansen and White Kernohan syndromes signature Reduced sensitivity may be observed.
Wolf-Hirschhorn syndrome & Rauch-Steindl syndrome	Chr4p16.3 deletion, *NSD2*	The range of validated coordinates is 4p16.3(679,715- 2,169,001). CNVs overlapping or expanding this region may also be detected. NSD2 sequence variants have been shown to match the episignature.
White-Sutton syndrome	*POGZ*	
Williams-Beuren syndrome	Chr7q11.23 deletion	CNVs overlapping or expanding 7q11.23 may also be detected.
Witteveen-Kolk syndrome	*SIN3A*	Reduced sensitivity may be observed.
Wieacker-Wolff syndrome	*ZC4H2*	Episignature defined with male cases only. Heterozygotes have been shown to not match the episignature. Reduced sensitivity may be observed.
Intellectual developmental disorder, X-linked 93	*BRWD3*	Healthy carriers and those with incomplete penetrance are detectable. Reduced sensitivity may be observed.
Intellectual developmental disorder, X-linked 97	*ZNF711*	Heterozygotes have been shown to match the episignature. Reduced sensitivity may be observed.
Klinefelter syndrome	ChrX duplication; 47,XXY	XXX cases may also be detected.
Hypermethioninemia with deficiency of S-adenosylhomocysteine hydrolase*	*AHCY*	Available as a single signature request only.
Diamond-Blackfan anemia 1*	*RPS19*	Available as a single signature request only. Reduced sensitivity against other Diamond-Blackfan anemia disorders may be observed.
Diamond-Blackfan anemia 5*	*RPL35A*	Available as a single signature request only. Reduced sensitivity against other Diamond-Blackfan anemia disorders may be observed.
Developmental delay with or without dysmorphic facies and autism*	*TRRAP*	Available as a single signature request only. Only for variants within the amino acid range of 960-1159.
Desanto-Shinawi syndrome*	*WAC*	Available as a single signature request only.
Hypercholesterolemia, familial, 1*	*LDLR*	Available as a single signature request only. Sensitivity against other hereditary

		hypercholesterolemia disorders has not been evaluated. Both monoallelic and biallelic cases are detected.
Intellectual developmental disorder with dysmorphic facies and behavioral abnormalities*	*FBXO11*	Available as a single signature request only.
KMT2C-related syndrome*	*KMT2C*	Available as a single signature request only. Reduced sensitivity and specificity may be observed.
Neurodevelopmental-craniofacial syndrome with variable renal and cardiac abnormalities*	*ZMYM2*	Available as a single signature request only.
PHF12-related syndrome*	*PHF12*	Available as a single signature request only.
SETD1A-related syndrome*	*SETD1A*	Available as a single signature request only.
Schuurs-Hoeijmakers syndrome*	*PACS1*	Available as a single signature request only. Reduced sensitivity may be observed.
Intellectual developmental disorder, X-linked 112*	*ZMYM3*	Available as a single signature request only. Episignature defined with male cases only. Reduced sensitivity and specificity may be observed.
Coffin-Siris syndrome 12*	*BICRA*	Available as a single request only. Reduced sensitivity and specificity may be observed.
TLK2-related Intellectual developmental disorder 57*	*TLK2*	Available as a single request only.
X-Linked PHF8-related Intellectual developmental disorder, Siderius type*	*PHF8*	Available as a single request only. Episignature defined with male cases only.
Neurofibromatosis, type 1*	*NF1*	Available as a single request only
NOTCH1-associated syndrome*	*NOTCH1*	Available as a single request only
Tessadori-Bicknell-van Haaften neurodevelopmental syndrome 1, 3 and 4*	*H4C4, H4C4, H4C5, H4C9*	Available as a single request only
Fragile X syndrome (Affected males ONLY)	*FMR1* promoter	Epivariant, based on specific regions.
Angelman syndrome	15q11.2-q13 (*SNRPN* promoter, *SNURF*)	Epivariant, based on specific regions.
Beckwith-Wiedemann syndrome	11p15.5 (*IC1* and *IC2*)	Epivariant, based on specific regions.
Kagami-Ogata syndrome	14q32 (*MEG3* promoter)	Epivariant, based on specific regions.
Mulchandani-Bhoj-Conlin syndrome	20q11-q13 (*GNAS*)	Epivariant, based on specific regions.
Multi-locus imprinting disturbances	All EpiSign imprinting regions	Epivariant, based on specific regions.
Pseudohypoparathyroidism IA & IB	20q11-q13 (*GNAS*)	Epivariant, based on specific regions.
Prader-Willi syndrome	15q11.2-q13 (*SNRPN*	Epivariant, based on specific regions.

	promoter, *SNURF*)	
Silver-Russell syndrome 1 & 2	11p15.5 (*IC1* and *IC2*), 7q32.2	Epivariant, based on specific regions.
Temple syndrome	14q32 (*MEG3* promoter)	Epivariant, based on specific regions.
Diabetes mellitus, transient neonatal 1	6q24 (*PLAGL1*)	Epivariant, based on specific regions.

Table S2. syndromes available at EpigenCentral and dataset used to derive the episignatures and classification models.

Disorder	Loci	Dataset	Array
Autism spectrum disorder	16p11.2 deletion	GSE113967	450k
	CHD8	GSE113967	450k
CHARGE syndrome	*CHD7*	GSE97362	450k
Down syndrome	chr21 trisomy	GSE52588	450k
Dup7 syndrome	7q11.23 duplication	GSE66552	450k
Kabuki syndrome	*KMT2D*	GSE97362	450k
Nicolaides-Baraitser syndrome	*SMARCA2*	GSE125367	EPIC
Sotos syndrome	*NSD1*	GSE74432	450k
Weaver syndrome	*EZH2*	GSE74432	450k
Williams syndrome	7q11.23 deletion	GSE66552	450k

Table S3: episignatures included in MethaDory (previous to the final release of their study), adapted from Ferraro et al., Supplementary Table 4.

Disorder	Loci	Signature	PMID
Helsmoortel-Van Der Aa Syndrome (HVDAS)	*ADNP*	Central	32109418
	ADNP	Central	31029150
	ADNP	Central	32758449
	ADNP	Central	35904121
	ADNP	Terminal	32109418
	ADNP	Terminal	31029150
	ADNP	Terminal	32758449
	ADNP	Terminal	35904121
KBG syndrome	*ANKRD11*, chr16q24.3del		36440975
Coffin-Siris syndrome	*ARID1A, ARID1B, SMARCB1, SMARCA4, SMARCA2*		32109418
BAFopathies (Coffin-Siris and Nicolaides-Baraitser)	*ARID1A, ARID1B*	c.6200	35904121
	ARID1A, ARID1B, SMARCB1, SMARCA4, SMARCA2		35904121
	SMARCA4	c2650	35904121
Bafopathy (Non-syndromic)	*ARID1A*	Duplication	39355979
	ARID1B	Duplication	39355979

Neurodevelopmental disorder with or without autism or seizures (NEDAUS)	*CUL3*		39501558
Bohring-Opitz syndrome	*ASXL1*		35361921
Alpha-thalassemia/Impaired intellectual development syndrome, X-linked	*ATRX*		32109418
	ATRX		35904121
BCL11B-related disease	*BCL11B*		37860968
Mental retardation (X-linked)	*BRWD3*		32109418
	BRWD3		35904121
Berardinelli-Seip Congenital Lipodystrophy type 2	BSCL2		36715159
Immunodeficiency with Centromeric instability and Facial anomalies (ICF) syndrome	*CDCA7, ZBTB24, HELLS*		32109418
	CDCA7, ZBTB24, HELLS		35904121
	CDCA7, ZBTB24, HELLS		33916664
CDK13-related disorder	*CDK13*		35063350
Epileptic encephalopathy	*CHD2*		32109418
	CHD2		39107278
	CHD2		35904121
Sifrim-Hitz-Weiss syndrome	*CHD4*		39824190
CHARGE syndrome	*CHD7*		32109418
	CHD7		28475860
	CHD7		35904121
Autism	*CHD8*		32109418
	CHD8		35904121
	CHD8		31311581
	chr16p11	Deletion	35904121
	chr16p11.2	Deletion	31311581
Down syndrome	chr21	Trisomy	32109418
	chr21	Trisomy	25701644
	chr21	Trisomy	35904121
	chr21	Trisomy	34440289
VeloCardioFacial syndrome	chr22q11.2	Deletion	35904121
	chr22q13.3	Deletion	35904121
	chr22q11.2	Deletion	34445317
	chr22q13.3	Deletion	33407854
Wolf-Hirschhorn syndrome	chr4p16.3	Deletion	35904121
Williams syndrome	chr7q11.23	Deletion	32109418
	chr7q11.23	Deletion	32303053
	chr7q11.23	Deletion	35904121
	chr7q11.23	Deletion	26166478
Dup7 Syndrome	chr7q11.23	Duplication	32109418
	chr7q11.23	Duplication	35904121
	chr7q11.23	Duplication	26166478
Rubinstein-Taybi syndrome	*CREBBP, EP300*		32109418
	CREBBP		35904121
	EP300		35904121
Menke-Hennekam syndrome	*CREBBP, EP300*	ID4	35904121
Intellectual developmental disorder, autosomal dominant 21	*CTCF*		38054406
Cerebellar ataxia, deafness and narcolepsy, autosomal dominant (ADCADN)	*DNMT1*		32109418
	DNMT1		35904121

Tatton-Brown-Rahman syndrome	*DNMT3A*	32109418
	DNMT3A	31160375
	DNMT3A	35904121
	DNMT3A	33916664
ICF1	*DNMT3B*	32109418
	DNMT3B	35904121
DOT1L associated syndrome	*DOT1L*	37827158
DYRK1A intellectual disability	*DYRK1A*	34345024
Cohen-Gibson syndrome	*EED*	35904121
Weaver syndrome	*EZH2*	35904121
	EZH2	32243864
Kleefstra syndrome	*EHMT1*	32109418
	EHMT1	10.20517/jtgg.2020.23
	EHMT1	35904121
9q34.3 microduplication syndrome	*EHMT1*	38384171
Intellectual developmental disorder, X-linked, syndromic, Armfield type (MRXSA)	*FAM50A*	33498634
	FAM50A	35904121
Fanconi anemia	*FANCA*	37865086
Rahman syndrome	*HIST1H1E*	32109418
	HIST1H1E	31910894
	HIST1H1E	35904121
Au-Kline syndrome	*HNRNPK*	36130591
Developmental and epileptic encephalopathy	*HNRNPU*	38797891
	HNRNPU	37120726
JARID2 neurodevelopmental syndrome	*JARID2*	37762546
	JARID2	35887345
Koolen-de-Vries syndrome	*KANSL1*	32109418
	KANSL1	38282074
	KANSL1	35904121
Arboleda-Tham syndrome	*KAT6A*	35904121
Genotiopatellar syndrome	*KAT6B*	32109418
	KAT6B	35904121
Say-Barber-Biesecker-Young-Simpson syndrome (Ohdo syndrome)	*KAT6B*	32109418
	KAT6B	35904121
KDM2B-related neurodevelopmental disorder	*KDM2B*	35904121
Autosomal dominant intellectual developmental disorder – 65	*KDM4B*	35904121
Claes-Jensen syndrome	*KDM5C*	32109418
	KDM5C	23356856
	KDM5C	35904121
	KDM5C	29456765
Wiedemann–Steiner syndrome	*KMT2A*	32109418
	KMT2A	35163737
	KMT2A	35904121
Dystonia 28, childhood-onset	*KMT2B*	34380541
	KMT2B	35904121
KMT2D-related syndrome	KMT2C	39013459

Kabuki syndrome	*KMT2D, KDM6A*		32109418
	KMT2D		28475860
	KMT2D, KDM6A		35904121
	KMT2D		33916664
Mental retardation (Autosomal dominant 51)	*KMT5B*		32109418
	KMT5B		35904121
MSL2-related NDDs	*MSL2*		38815585
Cornelia de Lange syndrome	*NIPBL, RAD2, SMC3, SMC1A*		32109418
	NIPBL, RAD21, SMC3, SMC1A		35904121
Sotos syndrome	*NSD1*		32109418
	NSD1		26690673
	NSD1		35904121
	NSD1		33916664
Hunter McAlpine syndrome	*NSD1*	Duplication	32109418
	NSD1	Duplication	35904121
Wolf-Hirschhorn syndrome	*NSD2*, 4q16.13		39669601
Böjer-son-Forssman-Lehmann syndrome	*PHF6*		32109418
	PHF6		35904121
Chung-Jansen syndrome	*PHIP*		38787418
Renpenning syndrome	*PQBP1*		35904121
PURA-related neurodevelopmental disorders	*PURA*		38770750
SETD1B-related syndrome	*SETD1B*		32109418
	SETD1B		31685013
	SETD1B		35904121
Luscan-Lumish syndrome	*SETD2*	c1740	37166351
	SETD2		35904121
	SETD2		33916664
Mental retardation (Autosomal dominant 23)	*SETD5*		35904121
Witteveen-Kolk syndrome	*SIN3A*		36399132
Nicolaides-Baraitser syndrrome	*SMARCA2*		31288860
Blepharophimosis intellectual disability syndrome (BIS)	*SMARCA2*		32694869
	SMARCA2		35904121
Blepharophimosis intellectual disability syndrome AND Helsmoortel-Van Der Aa Syndrome	*SMARCA2, ADNP*		38884529
Myopathy, lactic acido-sis and sideroblastic anemia 2	*YARS2*		35904121
Mental retardation (X-linked)	*SMS*		32109418
	SMS		35904121
Coffin-Siris syndrome	*SOX11*		35341651
	SOX11		35904121
Chromosome 1p36 deletion syndrome	*SPEN*		33596411
Floating-Harbor syndrome	*SRCAP*		32109418
	SRCAP		35904121
	SRCAP		33909990
	SRCAP	proximal	33909990
SRSF1-related syndrome	*SRSF1*		37071997
Pitt-Hopkins syndrome	*TCF4*		38571311

Beck-Fahrner syndrome	*TET3*		35904121
Clark-Baraitser syndrome	*TRIP12*		36430143
Mental retardation (X-linked)	*UBE2A*		32109418
	UBE2A		35904121
Hao-Fountain syn-drome	*USP7*		38126281
Recurrent constellations of embryonic malformations	VACTERL	combined	39089258
	VACTERL	intermediate	39089258
	VACTERL	robust	39089258
Fetal Valproate syndrome	Valproic Acid		39097820
Werner syndrome	*WRN, RECQL2*		28861129
Gabriele-de Vries syndrome	*YY1*		35904121
Mowat-Wilson syndrome	*ZEB2*		38351292
Developmental Delay with Gastrointestinal, Cardiovascular, Genitourinary, and Skeletal Abnormalities syndrome	*ZNF699*		39424669
Mental retardation (X-linked)	*ZNF711*		32109418
	ZNF711		35904121
ARID2-related disorder	*ARID2*		40044822

Table S4. Basic processing steps in each methylation study, ordered by date of release. All arrays eliminated Cross-reactive probes and those associated to SNP sites or which contained SNPs. In DMPs, if *methylation* is stated instead of M / Beta values, the article did not specify and was not deducible.

Title	Date release	Disease	Loci	Preprocessing	DMP or DMR call	Filters + ML
An Epigenetic Signature in Peripheral Blood Associated with the Haplotype on 17q21.31, a Risk Factor for Neurodegenerative Tauopathy	2014/03	Tauopathies (progressive supranuclear palsy (PSP) and frontotemporal dementia (FTD))		IDAT – Illumina genome studio.<br>Minfi (Swan normalization).<br>Batch correction using CoMBat.<br>XY probes included	DMP - Limma lm (Beta_vals ~ Diagnosis + blood cells (Houseman) + age + ethnicity + gender + array id)	p.Adj (FDR) < 0.05, deltaBeta > 0.1.<br>No ML model.
Identification of a DNA methylation signature in blood cells from persons with Down Syndrome	2015/02	Down syndrome		IDAT – Illumina genome studio.<br>IMA (preprocessing)	DMR: Manova (Beta_vals ~ Diagnosis + sex + batch + blood cells (Houseman))<br>DMPs: Anova (Beta_vals ~ Diagnosis + sex + batch + blood cells (Houseman))	No ML model.
Genome-wide methylation analysis in Silver-Russell syndrome patients	2015/03	Silver-Russell	*ICR1*, mUPD, other	IDAT. Quantile normalization without further filtering.	DMP - Study intensity changes (methylated and unmethylated)	No ML model.
Symmetrical Dose-Dependent DNA-Methylation Profiles in Children with Deletion or Duplication of 7q11.23	2015/08	Williams		IDAT – Illumina genome studio.<br>XY filtered out.	DMP - Limma lm (Beta_vals ~ Diagnosis + blood cells (Houseman))	No ML model.
NSD1 mutations generate a genome-wide DNA methylation signature.	2015/12	Sotos	*NSD1*	IDAT – Illumina genome studio.<br>XY kept.	Mann-Whitney Utest	pAdj (Bonf) > 0.05, deltaBeta > 0.2<br>No ML model.
Identification of a methylation profile for DNMT1-associated autosomal dominant	2016/09	adult-onset autosomal dominant	*DNMT1*	IDAT – Genome studio.	DMRs - Partek Genomics Suite, using an ANOVA test.	pval < 0.01, mean F-val > 50 and deltaBeta > 0.2 in 5 consecutive CpGs.

cerebellar ataxia, deafness, and narcolepsy		cerebellar ataxia with deafness and narcolepsy				No ML model.
Clinical Validation of Fragile X Syndrome Screening by DNA Methylation Array	2016/11	Fragile X syndrome	*FMR1*	IDAT – Genome studio.	DMRs - Partek Genomics Suite, using an ANOVA test.	pval < 0.01, mean F-val > 50 and deltaBeta > 0.15 in 3 consecutive CpGs. No ML model.
The defining DNA methylation signature of Floating-Harbor Syndrome	2016/12	Floating-Harbor	*SRCAP*	IDAT – Genome studio.	DMRs - Partek Genomics Suite, using an ANOVA test.	pval < 0.01, mean F-val > 50 and deltaBeta > 0.2 in 5 consecutive CpGs. No ML model.
Identification of epigenetic signature associated with alpha thalassemia/mental retardation X-linked syndrome	2017/03	Alpha-thalassemia and X-linked mental retardation	*ATRX*	IDAT – Genome studio.	DMRs - Partek Genomics Suite, using an ANOVA test.	pval < 0.01, mean F-val > 50 and deltaBeta > 0.2. in 4 consecutive CpGs No ML model.
Genome-wide multilocus imprinting disturbance analysis in Temple syndrome and Kagami-Ogata syndrome	2017/04	Temple syndrome and Kagami-Ogata syndrome	14q32.2 region	IDAT – GenomeStudio. XY ruled out. Limma (Quantile Normalization). Batch correction using ComBat.	DMP - Test beta-value difference at average and standard-deviation level between conditions. If above 3SD, significant and a minimum of deltaBeta > 0.05.	No ML model.
CHARGE and Kabuki Syndromes: Gene-Specific DNA Methylation Signatures Identify Epigenetic Mechanisms Linking These Clinically Overlapping Conditions	2017/05	CHARGE	*CHD7*	IDAT – GenomeStudio.	DMP - Limma (M_vals ~ Diagnosis)	P.val < 0.01 & deltaBeta > 0.1. Model using kernlab (SVM)
		Kabuki	*KMT2D*	IDAT – GenomeStudio.	DMP - Limma (M_vals ~ Diagnosis)	P.val < 0.01 & deltaBeta > 0.1. Model using kernlab (SVM)
Genome-wide DNA methylation analysis in blood cells from patients with Werner syndrome	2017/08	Werner syndrome	*WRN*	IDAT – minfi (functional normalization).	DMP – ANOVA.	P.val (non.adj) < 0.001 & deltaBeta > 0.3

					DMR – MANOVA (significance in, at least, 3 consecutive CpG)	No ML model
Patients with a Kabuki syndrome phenotype demonstrate DNA methylation abnormalities	2017/11	Kabuki	*KMT2D, KMT2A, HCFC1, ZBTB24, KMT2B and DMT3B*	IDAT – minfi (Quantile normalization). Eliminate non-cordant sex samples.	DMPs - limma (M_vals ~ Diagnosis + blood cells (Houseman) + ancestry)<br>DMRs – Bumphunter	No ML model.
The defining DNA methylation signature of Kabuki syndrome enables functional assessment of genetic variants of unknown clinical significance	2017/12	Kabuki	*KMT2D*	IDAT – minfi (Illumina normalization).<br>XY probes ruled out.	DMPs - limma (M_vals ~ Diagnosis + blood cells (Houseman))<br>DMRs – Bumphunter (FWER < 0.01)	P.val (FDR) < 0.01 & deltaBeta > 0.1, CpGs with AUC > 0.95 and R^2 correlation < 0.95.<br><br>e1071 (Multiclass SVM radial, 10fold cv)
Genomic DNA Methylation Signatures Enable Concurrent Diagnosis and Clinical Genetic Variant Classification in Neurodevelopmental Syndromes	2018/01	Neurodevelopmental syndromes (14 in total)		IDAT – minfi (Illumina normalization).<br>XY probes ruled out.<br>Eliminate non-cordant sex samples.	DMPs - limma (M_vals ~ Diagnosis + blood cells (Houseman))<br>DMRs – Bumphunter	P.val (FDR) < 0.01 & deltaBeta > 0.1, CpGs with AUC > 0.8 and R^2 correlation < 0.8.<br>e1071 (SVM linear, 10fold cv)
Peripheral blood epi-signature of Claes-Jensen syndrome enables sensitive and specific identification of patients and healthy carriers with pathogenic mutations in KDM5C	2018/02	Claes-Jensen	*KDM5C*	IDAT – minfi (Illumina normalization).<br>XY probes ruled out.<br>Eliminate non-cordant sex samples.	DMPs - limma (M_vals ~ Diagnosis + blood cells (Houseman))<br>DMRs – Bumphunter	P.val (FDR) < 0.01 & deltaBeta > 0.1, CpGs with AUC = 1 and R^2 correlation < 0.8.<br>e1071 (SVM radial, 10fold cv)

Primary constitutional MLH1 epimutations: a focal epigenetic event	2018/04		*MLH1*	IDAT – Genome Studio RnBeads (batch effect correction)	DMP – RnBeads	No ML model
Identification of rare de novo epigenetic variations in congenital disorders	2018/05	Mix of unidentified cases		Raw betas – lumi/methyllumi (BMIQ normalization) X chr CpG analysed independently. Eliminate non-cordant sex samples.	DMR – sliding window (at least 3 CpGs with beta-vals above 99.9th percentile compared with controls, a mean deltaBeta 0.15 and at least 1 above 0.1 deltaBeta.	No ML model.
Comparative methylome analysis of ICF patients identifies heterochromatin loci that require ZBTB24, CDCA7 and HELLS for their methylated state	2018/07	ICF syndrome	*DNMT3B* (ICF1), *ZBTB24* (ICF2), *CDCA7* (ICF3), *HELLS* (ICF4)	IDAT – Genome Studio Quantile normalization. XY chr CpG analysed independently.	DMP – median deltaBeta > 0.2	No ML model.
BAFopathies' DNA methylation epi-signatures demonstrate diagnostic utility and functional continuum of Coffin–Siris and Nicolaides–Baraitser syndromes	2018/11	Coffin-Siris and Nicolaides-Baraitser	*ARID1B*, *SMARCB1* (CFSS), *SMARCA2* (NCBS)	IDAT – minfi (Illumina normalization). XY probes ruled out.	DMPs - limma (M_vals ~ Diagnosis + blood cells (Houseman)) DMRs – DMRcate	P.val (FDR) < 0.05 & deltaM > 5% (top 1000 CpGs), CpGs with AUC > 0.85 and R^2 correlation < 0.85. e1071 (SVM radial) 10fold cv. Separate cohort in 75% training and 25% testing.

Diagnostic Utility of Genome-wide DNA Methylation Testing in Genetically Unsolved Individuals with Suspected Hereditary Conditions	2019/03	Mix		IDAT – minfi (Illumina normalization). XY probes ruled out.	DMPs - limma (M_vals ~ Diagnosis + blood cells (Houseman)) DMRs – bumphunter	deltaBeta > varied threshold (max for Sotos, minimum for BAFopathies, CdLS and Charge), order by P.val and (top 1000 CpGs), CpGs with AUC > 0.8 and R^2 correlation < 0.8. e1071 (SVM linear) 10fold cv. Separate cohort in 75% training and 25% testing.
Gene domain-specific DNA methylation episignatures highlight distinct molecular entities of ADNP syndrome	2019/04	Helsmoortel-van der Aa syndrome	*ADNP*	IDAT – minfi (Illumina normalization). XY probes ruled out. Eliminate non-cordant sex samples.	DMPs - limma (M_vals ~ Diagnosis + blood cells (Houseman)) DMRs – DMRcate	P.val (FDR) < 0.01 & deltaBeta > 0.1, CpGs with AUC > 0.85 and R^2 correlation < 0.85. e1071 (SVM radial) 10fold cv. Separate cohort in 75% training and 25% testing.
DNA methylation abundantly associates with fetal alcohol spectrum disorder and its subphenotypes	2019/05	FASD		IDAT MethylAid (minfi). Quantile normalization. XY removed.	Minfi::DMPs (M_vals, blood cells(minfi)) DMRs – bumphunter.	No ML model.
Functional DNA methylation signatures for autism spectrum disorder genomic risk loci: 16p11.2 deletions and CHD8 variants	2019/07	Autism Spectrum Disorder	16p11.2del and CHD8	IDAT – minfi (Illumina normalization). XY probes ruled out. Eliminate non-cordant sex samples.	DMPs – Combination of qlucore for site selection, limma (M_vals ~ Diagnosis + age + sex + blood cells (Houseman)) for coovariates and Mann-Whitnet U test (for non-parametric effects) DMRs – Bumphunter.	Limma (p.val (FDR) > 0.05 & deltaBeta > 0.05), Mann-Whitney (p.val (FDR) > 0.05). Significant sites were their intersection. Build classification scores as medianCPG value (control and case): (r(Value CpG, median case profile) - (Value CpG, median control profile)

Screening for rare epigenetic variations in autism and schizophrenia	2019/07	Autism and Schizophrenia		IDAT - lumi (BMIQ normalization). Outlyer removal based on PC1 and PC2	DMRs – sliding window to detect epimutations. 1kb length, minimum 3 CpGs and with beta value above the 99.9th percentile compared to controls, and a deltaBeta > 0.15.	No ML model.
Growth disrupting mutations in epigenetic regulatory molecules are associated with abnormalities of epigenetic aging	2019/07	Tatton-Brown syndrome	*DNMT3A*	IDAT – wateRmelon (dasen function). XY probes removed	DMPs – limma (methylation ~ Diagnosis + Sex) DMRs - DMRcate	P.val (BH) < 0.05
New insights into DNA methylation signatures: SMARCA2 variants in Nicolaides-Baraitser syndrome	2019/09	Nicolaides-Baraitser	*SMARCA2*	IDAT – minfi (Illumina normalization). XY probes ruled out.	DMPs - limma (M_vals ~ Diagnosis + age + sex + monocytes proportion (Houseman)) DMRs – DMRcate	P.val (FDR) < 0.05 & deltaBeta > 0.05. Build classification scores as medianCPG value (control and case): (r(Value CpG, median case profile) - (Value CpG, median control profile)
An epigenetic biomarker for adult high-functioning autism spectrum disorder	2019/09	High Functioning ASD		IDAT – ChAMP (BMIQ normalization). XY probes ruled out. ComBat to correct batches (Sex and Array)	DMP - Limma	randomForest (random forest, 10fold CV) and CALF (Coarse Approximation Linear Function, 2000 repeats). Evaluation metric: ROC.
Epigenetic signatures of Werner syndrome occur early in life and are distinct from normal epigenetic aging processes	2019/10	Werner syndrome	*WRN, LMNA, POLD1*	IDAT – minfi (quantile normalization). XY probes ruled out.	DMP - Limma (methylation ~ Diagnosis + age + sex + blood cells (houseman)) DMR – comb-p package based on limma p-values.	Pval (adjusted) < 0.05
A genome-wide DNA methylation signature for SETD1B-related syndrome	2019/11	Microdeetion 12q31.24	*SETD1B, KDM2B*	IDAT – minfi (Funnorm normalization).	DMP – minfi (M_vals ~ Diagnosis + age + sex + batch+	P.val (FDR) < 0.05 and R^2 < 0.8. No ML model.

				XY probes ruled out.	blood cells (FlowSorted.Blood.EPIC))	
Frameshift mutations at the C-terminus of HIST1H1E result in a specific DNA hypomethylation signature	2020/01	Rahman	*HIST1H 1E*	IDAT – minfi (Illumina normalization). XY probes ruled out.	DMPs - limma (M_vals ~ Diagnosis + blood cells (Houseman)) DMRs – DMRcate	P.val (FDR) < 0.001 & deltaBeta > 0.1. Caret (Random Forest), 10fold CV
Evaluation of DNA Methylation Episignatures for Diagnosis and Phenotype Correlations in 42 Mendelian Neurodevelopmental Disorders	2020/02	Mix		IDAT – minfi (Illumina normalization). XY probes ruled out. Different thresholds for each syndrome.	DMPs - limma (M_vals ~ Diagnosis + blood cells (Houseman) + top10 PC (if necessary))	Order and select top (-log(Pval) * deltaMethy) and AUC > threshold and R^2 < threshold. e1071 (SVM linear). Separate cohort in 75% training and 25% testing, except for syndromes with 10 or less cases.
DNA Methylation Signature for EZH2 Functionally Classifies Sequence Variants in Three PRC2 Complex Genes	2020/04	Weaver syndrome	*EZH2*	IDAT - Minfi. XY probes ruled out.	DMPs - limma (M_vals ~ Diagnosis + blood cells (FlowSorted.Blood)) DMRs – Bumphunter	P.val (FDR) < 0.05 & deltaBeta > 0.1. Build classification scores as medianCPG value (control and case): (r(Value CpG, median case profile) - (Value CpG, median control profile) Train caret (SVM).
EHMT1 pathogenic variants and 9q34.3 microdeletions share altered DNA methylation patterns in patients with Kleefstra syndrome	2020/06	Kleefstra syndrome	*EHMT1*, 9q34.3	IDAT – Minfi (Illumina normalization). XY probes ruled out. PCA for batches	DMPs – limma (methylation ~ sex + age + batch + blood cells (houseman)).	P.val (FDR) < 0.05 & deltaBeta > 0.1, r^2 < 0.9 SVM (caret).
De novo SMARCA2 variants clustered outside the helicase domain cause a new recognizable syndrome with intellectual disability	2020/07		*SMARC A2* (outside	IDAT – minfi (Illumina normalization).	DMPs - limma (M_vals ~ Diagnosis + blood cells (Houseman))	P.val (FDR) < 0.05 & deltaBeta > 0.1. No model.

and blepharophimosis distinct from Nicolaides–Baraitser syndrome			helicase domains)	XY probes ruled out. Discordant sex samples discarded. PCA for batches and outlyers.	DMRs – DMRcate	
Integrated DNA methylation analysis reveals a potential role for ANKRD30B in Williams syndrome	2020/09	Williams	7q11.23del	IDAT - ChAMP (BMIQ normalization). ComBat to correct batches (age, sex, array)	DMPs - limma (beta_vals ~ Diagnosis) DMRs – DMRcate. WGCNA to find co-methylation modules.	P.val (FDR) < 0.05 & deltaBeta > 0.1. No ML model.
Episignatures Stratifying Helsmoortel-Van Der Aa Syndrome Show Modest Correlation with Phenotype	2020/09	Helsmoortel-Van Der Aa Syndrome	*ADNP*	IDAT – Not specified	DMPs - Lm(Methylation ~ Diagnosis + Age + Sex + CD4T + natural killer cells + B cells + Monocytes + Granulocytes + Batch)	No ML model.
Genome-wide methylation analysis in Silver–Russell syndrome, Temple syndrome, and Prader–Willi syndrome	2020/10	Imprinting disorders (Silver–Russell syndrome, Temple syndrome and Prader–Willi syndrome)		IDAT - ChAMP (BMIQ normalization). ComBat to correct batches (Sex and Age)	DMPs – ChAMP (beta_vals ~ Diagnosis). DMRs - at least two consecutive probes within iDMRs (formed by 4 or more probes) or at least three consecutive probes within 2000 bp from a transcription starting site.	P.val (FDR) < 0.05 & deltaBeta > 0.1. No ML model.
Differential methylation of imprinting genes and MHC locus in 22q11.2 deletion syndrome-related schizophrenia spectrum disorders	2021/01	22q11.2 Deletion Syndrome	22q11.2del	IDAT - ChAMP (BMIQ normalization). XY probes ruled out ComBat to correct batches (Array)	DMPs – ChAMP DMRs – ChAMP	No ML model.

DNA methylation epi-signature is associated with two molecularly and phenotypically distinct clinical subtypes of Phelan-McDermid syndrome	2021/01	Phelan-McDermid	*22q13.3 del* , *SHANK3*	IDAT – Minfi (Illumina normalization).<br>XY probes ruled out.<br>PCA for outlier removal.	DMPs - limma (M_vals ~ Diagnosis + blood cells (Houseman))<br>DMRs – Bumphunter DMRcate	P.val (FDR) < 0.01 & deltaBeta > 0.1.<br>Order and select top (-log(Pval) * deltaMethy) and AUC > threshold and R^2 < threshold.<br>e1071 (SVM linear). 10fold CV.
SPEN haploinsufficiency causes a neurodevelopmental disorder overlapping proximal 1p36 deletion syndrome with an episignature of X chromosomes in females	2021/03	1p36 deletion syndrome	*SPEN*	IDAT – Minfi (Illumina normalization).<br>XY probes ruled out (X chr probes used alone)	DMPs - limma (M_vals ~ Diagnosis + blood cells (Houseman))	Order and select top 1000 (-log(Pval) * deltaMethy) and top 500 AUC and R^2 < 0.9.<br>e1071 (SVM linear).
Truncating SRCAP variants outside the Floating-Harbor syndrome locus cause a distinct neurodevelopmental disorder with a specific DNA methylation signature	2021/04	Atypical FLHS phenotype	*SRCAP*	IDAT – Minfi (Illumina normalization).<br>XY probes ruled out.	DMPs - limma (M_vals ~ Diagnosis + Sex + Age + Batch + blood cells except neutrophyls (Houseman))	P.val (FDR) < 0.01 & deltaBeta > 0.2.<br>Caret (SVM linear)
Interplay between Histone and DNA Methylation Seen through Comparative Methylomes in Rare Mendelian Disorders	2021/04	ICF<br>Sotos<br>Tatton-Brown<br>Kabuki<br>Luscan Lumish	*DNMT3B*<br>*NSD1*<br>*DNMT3A*<br>*KMT2D*<br>*SETD2*	IDAT – ChAMP (BMIQ normalization).<br>XY probes ruled out.<br>ChAMP.Combat for batches.	DMPs – ChAMP.limma (Beta_vals ~ Diagnosis)	P.val (BH) < 0.05
PRDX1 gene-related epi-cblC disease is a common type of inborn error of cobalamin metabolism with mono- or bi-allelic MMACHC epimutations	2021/07	epi-cblC disease	*PRDX1, MMACHC* epimutation	IDAT – GenomeStudio and minfi (SWAN normalization).	DMPs – t.test between cases and controls.	No ML model.

Childhood-onset dystonia-causing KMT2B variants result in a distinctive genomic hypermethylation profile	2021/08	Dystonia	*KMT2B*	IDAT – Minfi (Illumina normalization). XY probes ruled out.	DMPs - limma (M_vals ~ Diagnosis + blood cells (Houseman))	Order and select top 1000 (-log(Pval) * deltaMethy) and top 500 AUC and $R^2 < 0.9$. e1071 (SVM linear)
Identification of a DNA Methylation Episignature in the 22q11.2 Deletion Syndrome	2021/08	22q11.2 Deletion Syndrome		IDAT – Minfi (Illumina normalization). XY probes ruled out. PCA for batch removal.	DMPs - limma (M_vals ~ Diagnosis + blood cells (Houseman)) DMRs – DMRcate	Order and select top 900 (-log(Pval) * deltaMethy) and top 450 AUC and $R^2 < 0.6$. e1071 (SVM linear) leave-one-out CV and leave-25%-out CV.
Deficiency of TET3 leads to a genome-wide DNA hypermethylation episignature in human whole blood	2021/11	TET3 defficiency	*TET3*	IDAT – Minfi (Illumina normalization). XY probes ruled out. PCA for batch removal.	DMPs - limma (M_vals ~ Diagnosis + blood cells (Houseman)) DMRs – DMRcate	P.val (FDR) < 0.001 and deltaBeta > 0.1. top AUC and $R^2 < 0.8$. e1071 (SVM linear) 10fold CV.
Integrative approach to interpret DYRK1A variants, leading to a frequent neurodevelopmental disorder	2021/11	DYRK1A intellectual disability	*DYRK1A*	IDAT – minfi (Illumina normalization). XY probes ruled out.	DMPs - limma (M_vals ~ Diagnosis + age + sex + blood cells (Houseman))	P.val (BH) < 0.05 & deltaBeta > 0.1. SVM (linear kernel)
Novel diagnostic DNA methylation episignatures expand and refine the epigenetic landscapes of Mendelian disorders.	2022/01	MultiClass		IDAT – Minfi (Illumina normalization). XY probes ruled out.	DMPs - limma (M_vals ~ Diagnosis + blood cells (Houseman)) DMRs – DMRcate	deltaBeta > 0.05. Order and select top (-log(Pval) * deltaMethy) and top AUC and R^2 (different thresholds). e1071 (SVM linear). 4foldCV.
Clinical Utility of a Unique Genome-Wide DNA Methylation Signature for KMT2A-Related Syndrome	2022/02	Wiedemann–Steiner	*KMT2A*	DAT – Minfi (Illumina normalization). XY probes ruled out. PCA for outlyers (samples outside 3SD discarded)	DMPs - limma (M_vals ~ Diagnosis + blood cells (Houseman)) DMRs – DMRcate	deltaBeta > 0.05. Order and select top 500 (-log(Pval) * deltaMethy) and top 250 AUC and R^2 < 0.85 e1071 (SVM linear). 10foldCV.

DNA methylation episignature in Gabriele-de Vries syndrome	2022/04	Gabriele-de-Vries	*YY1*	DAT – Minfi (Illumina normalization). XY probes ruled out. PCA for outlyers and batch effects.	DMPs - limma (Beta_values ~ Diagnosis + blood cells (Houseman))	deltaBeta > 0.05. Order and select top 1000 (-log(Pval) * deltaMethy) and top AUC and R^2. e1071 (SVM linear). 10foldCV. Separate cohort in 75% training and 25% testing.
Blood DNA methylation provides an accurate biomarker of KMT2B-related dystonia and predicts onset	2022/04	KMT2B-related dystonia	*KMT2B*	IDAT – Minfi (Quantile normalization). XY probes ruled out.	DMPs - limma (M_vals ~ Diagnosis + Sex + age + blood cells (Houseman)) DMRs – DMRcate	P.val < 5e-8 and logFC > 1. e1071 (SVM linear). 10fold CV.
Epimutations in both the TESK2 and MMACHC promoters in the Epi-cblC inherited disorder of intracellular metabolism of vitamin B12	2022/04	Epi-cblC	*TESK2* and *MMACHC* EpiMutations	IDAT – GenomeStudio and minfi (SWAN normalization). PCA for batches and outliers.	DMPs – T.test for beta values (SNP & Variation Suite, Golden HELIX)	No ML Model
CDK13-related disorder: Report of a series of 18 previously unpublished individuals and description of an epigenetic signature	2022/05	CDK13-related disorder (CDK13-RD)	*CDK13*	IDAT – Minfi (Illumina normalization). XY probes ruled out.	DMPs - limma (M_vals ~ Diagnosis + blood cells (Houseman)) DMRs – DMRcate	deltaBeta > 0.05. Order and select top 700 (-log(Pval) * deltaMethy) and top 140 AUC. e1071 (SVM linear). Separate cohort in 75% training and 25% testing.
Comparison of Methylation Episignatures in KMT2B- and KMT2D-Related Human Disorders	2022/05	Kabuki and Dystonia28	*KMT2B, KMT2D*	FASTQ – TrimGalore (filters) and Bismark (alignment and methylation call). RnBeads (10x minimum). XY probes ruled out.	DMPs – RnBeads limma. DMRs – RnBeads DMRs.	No ML Model.

				PCA for batches and outlyers. Batch correction using ComBat.		
DNA methylation signature associated with Bohring-Opitz syndrome: a new tool for functional classification of variants in ASXL genes	2022/06	Bohring-Opit	*ASXL1*	IDAT – Minfi (Illumina normalization). XY probes ruled out.	DMPs - limma (Beta_vals ~ Diagnosis + blood cells except Neutrophils (Houseman) + epigenetic age (Horvath))	P.val (FDR) < 0.05, deltaBeta > 0.1. $R^2 < 0.9$. caret (SVM linear).
SOX11 variants cause a neurodevelopmental disorder with infrequent ocular malformations and hypogonadotropic hypogonadism and with distinct DNA methylation profile	2022/06	CSS9	*SOX11*	IDAT – Minfi (Illumina normalization). XY probes ruled out.	DMPs - limma (Beta_vals ~ Diagnosis + blood cells (Houseman)).	deltaBeta > 0.05. Order and select top 1000 (-log(Pval) * deltaMethy) and top 500 AUC and $R^2 < 0.6$. e1071 (SVM linear). Separate cohort in 75% training and 25% testing.
DNA Methylation Signature for JARID2-Neurodevelopmental Syndrome	2022/07	JARID2-syndrome	*JARID2*	IDAT – Minfi (Illumina normalization). XY probes ruled out. PCA for outliers and batches.	DMPs - limma (M_vals ~ Diagnosis + blood cells (Houseman)). DMRs – DMRcate.	deltaBeta > 0.05. Order and select top 1000 (-log(Pval) * deltaMethy) and top 250 AUC and $R^2 < 0.9$. e1071 (SVM linear). Separate cohort in 75% training and 25% testing.
An HNRNPK-specific DNA methylation signature makes sense of missense variants and expands the phenotypic spectrum of Au-Kline syndrome	2022/09	Au-Kline	*HNRNPK*	IDAT – Minfi. XY probes ruled out. PCA for outliers and batches.	DMPs - limma (M_vals ~ Diagnosis + blood cells (FlowSorted.Blood.EPIC)).	P.val (FDR) < 0.05, deltaBeta > 0.1. Build classification scores as medianCPG value (control and case): (r(Value CpG,

						median case profile) - (Value CpG, median control profile)
Episignature Mapping of TRIP12 Provides Functional Insight into Clark-Baraitser Syndrome	2022/11	Clark-Baraitser	*TRIP12*	IDAT – Minfi (Illumina normalization). XY probes ruled out. PCA for outliers and batches.	DMPs - limma (M_vals ~ Diagnosis + blood cells (Houseman)). DMRs – DMRcate.	deltaBeta > 0.05. Order and select top 1000/900 (-log(Pval) * deltaMethy) and top 300/500 AUC and $R^2 < 0.6$. e1071 (SVM linear). Separate cohort in 75% training and 25% testing. 4fold cv.
DNA methylation episignature for Witteveen-Kolk syndrome due to SIN3A haploinsufficiency	2022/11	Witteveen-Kolk syndrome	*SIN3A*	IDAT – Minfi XY probes ruled out. Discordant sex discarded. PCA for outliers and batches.	DMPs - limma (M_vals ~ Diagnosis + blood cells (Houseman)). DMRs – DMRcate.	deltaBeta > 0.05. Order and select top (-log(Pval) * deltaMethy) and top AUC and R^2. e1071 (SVM linear). Separate cohort in 75% training and 25% testing. 4fold CV.
Functional correlation of genome-wide DNA methylation profiles in genetic neurodevelopmental disorders	2022/11	Mix		IDAT – Minfi (Illumina normalization). XY probes ruled out. Probes with beta values of 0 and top 1% variable removed.	DMP – limma (beta_vals ~ Diagnosis + blood cells (houseman). DMRs – DMRcate	Pval adj < 0.01 & deltaBeta > 0.5. (In some cohort, non-adjusted value < 0.001)
The discovery of the DNA methylation episignature for Duchenne muscular dystrophy	2023/01	Duchenne muscular distrophy	*DMD*	IDAT – Minfi (Illumina normalization) XY probes ruled out. Discordant sex discarded. PCA for outliers and batches.	DMPs - limma (M_vals ~ Diagnosis + blood cells (Houseman)). DMRs – DMRcate.	deltaBeta > 0.05. Order and select top 1000 (-log(Pval) * deltaMethy) and top AUC > 0.8 and $R^2 < 0.8$. e1071 (SVM linear). Separate cohort in 75% training and 25% testing. 10fold CV.

Delineation of a KDM2B-related neurodevelopmental disorder and its associated DNA methylation signature	2023/01	KDM2B-related neurodevelopmental disorder	*KDM2B*	IDAT – Minfi (Illumina normalization)<br>XY probes ruled out.<br>Discordant sex discarded.<br>PCA for outliers and batches.	DMPs - limma (M_vals ~ Diagnosis + blood cells (Houseman)).	deltaBeta > 0.05. Order and select top 1000 (-log(Pval) * deltaMethy) and top AUC and R^2.<br>e1071 (SVM linear). Separate cohort in 75% training and 25% testing. 10fold CV.
Detection of a DNA Methylation Signature for the Intellectual Developmental Disorder, X-Linked, Syndromic, Armfield Type	2023/01	intellectual developmental disorder, X-linked, syndromic, Armfield type (MRXSA)	*FAM50A*	IDAT – Minfi (Illumina normalization)<br>XY probes ruled out.<br>Discordant sex discarded.<br>PCA for outliers and batches.	DMPs - limma (M_vals ~ Diagnosis + blood cells (Houseman)).<br>DMRs – DMRcate	deltaBeta > 0.05. Order and select top 1000 (-log(Pval) * deltaMethy) and top 500 AUC and $R^2 > 0.95$.<br>e1071 (SVM linear). Separate cohort in 75% training and 25% testing. 10fold CV.
Altered DNA methylation and gene expression predict disease severity in patients with Aicardi-Goutières syndrome	2023/04	Aicardi-Goutières	*RNASE H2B*	IDAT – Minfi and ChAMP (Quantile normalization and BMIQ)<br>ChAMP – CoMBat for batch effects (Age and Gender)	DMPs - limma (M_vals ~ Diagnosis).<br>DMRs – DMRcate	Pvalue (FDR) < 0.05 & deltaBeta > 0.1.
ANKRD11 pathogenic variants and 16q24.3 microdeletions share an altered DNA methylation signature in patients with KBG syndrome	2023/04	KBG syndrome	*ANKRD 11*, 16p24.3	IDAT – MINFI. XY probes ruled out.	DMPs – limma (methylation ~ Diagnosis + Age + Sex + blood cells (houseman) + SV1	Pvalue (FDR) < 0.05 & deltaBeta > 0.1.<br><br>SVM (caret)
Epigenotype–genotype–phenotype correlations in SETD1A and SETD2 chromatin disorders	2023/05	SETD1A and SETD2 chromatin disorders	*SETD1, SETD2*	FASTQ – TrimGalore (filters) and Bismark (alignment and methylation call).	DMPs – RnBeads limma implementation (M_vals ~ Diagnosis + Age + Batch + Sex + blood cells (Houseman))	Pvalue (FDR) < 0.05 & deltaBeta > 0.2.<br>No ML Model.

				RnBeads (10x minimum). XY probes ruled out.<br>PCA for batches and outlyers.<br>Batch correction using ComBat.		
Expression Quantitative Trait Methylation Analysis Identifies Whole Blood Molecular Footprint in Fetal Alcohol Spectrum Disorder (FASD)	2023/05	FASD		IDAT MethylAid (minfi).<br>Noob normalization.<br>XY removed.	Minfi::DMPs (Beta_vals, blood cells(minfi))<br><br>DMRs – Curstom. Consecutive significant DMP within 1000bp. DMRs at least 2 DMPs.	Pvalue (FDR) < 0.05<br><br>No ML Model.
DNA methylation epi-signature and biological age in attention deficit hyperactivity disorder patients	2023/05	Attention deficit hyperactivity disorders (ADHD)		IDAT – Minfi (Quantile normalization)<br>XY probes ruled out.	DMPs – decideTests limma function to compare cases and controls.<br>DMRs - DMRcate	Pvalue (adj) < 0.05.<br>No ML Model
Accelerated epigenetic aging and DNA methylation alterations in Berardinelli–Seip congenital lipodystrophy	2023/05	Berardinelli–Seip congenital lipodystrophy type 2 (CGL2)	*BSCL2*	IDAT - RnBeads. Greedycut and filtering.blacklist option for filtering	DMPs – limma (methylation ~ age + sex + blood cells (houseman) + SV).<br>DMRs – based on significant CpGs	Pvalue (adj) < 0.05
SRSF1 haploinsufficiency is responsible for a syndromic developmental disorder associated with intellectual disability	2023/05	SRSF1-related syndrome	*SRSF1*	IDAT – Minfi (Illumina normalization)<br>XY probes ruled out.<br>PCA for outliers and batches.	DMPs - limma (M_vals ~ Diagnosis + blood cells (Houseman)).<br>DMRs – DMRcate	Order and select top 800 (-log(Pval) * deltaMethy) and top AUC 267 and $R^2 < 0.6$<br><br>e1071 (SVM linear). Separate cohort in 75% training and 25% testing
Germline pathogenic variants in HNRNPU are associated with alterations in blood methylome	2023/07	developmental and epileptic	*HNRNPU*	FASTQ – TrimGalore (filters) and Bismark (alignment and methylation call).	DMPs – RnBeads limma implementation (M_vals ~ Diagnosis + Age + Batch + Sex	Pvalue (adj) < 0.01 and deltaBeta > 0.2.<br>No ML Model.

		encephalopathy 54		RnBeads (10x minimum). XY probes ruled out.<br>PCA for batches and outlyers.<br>Batch correction using ComBat (Age, gender and Batch).	+ blood cells (Houseman)) or two-sided Welch test.<br>DMRs – cluster significant close DMPs.	
DNA methylation episignature and comparative epigenomic profiling of HNRNPU-related neurodevelopmental disorder	2023/08	developmental and epileptic encephalopathy 54	*HNRNPU*	IDAT – Minfi (Illumina normalization)<br>XY probes ruled out. PCA for outliers and batches.	DMPs - limma (M_vals ~ Diagnosis + blood cells (Houseman)).<br>Process repeated 3 times with different subsampling.<br>DMRs – DMRcate	deltaBeta > 0.05. Order and select top 900-1000 (-log(Pval) * deltaMethy) and top AUC and R^2 < 0.65-0.7.<br>e1071 (SVM linear). 4fold CV.
Identification of a DNA methylation signature for Renpenning syndrome (RENS1), a spliceopathy)	2023/08	Renpenning syndrome (RENS1)	*PQBP1*	IDAT – Minfi (Illumina normalization)<br>XY probes ruled out. PCA for outliers and batches.	DMPs - limma (M_vals ~ Diagnosis + blood cells (Houseman)).<br>DMRs – DMRcate	deltaBeta > 0.05. Order and select top 800 (-log(Pval) * deltaMethy) and top 160 AUC and R^2 < 0.85.<br>e1071 (SVM linear).
Episignature analysis of moderate effects and mosaics	2023/09	Kabuki	*KMT2B, KMT2D*	IDAT – Minfi (Quantile normalization)<br>XY probes ruled out. PCA for outliers and batches, keep samples within 99 CI ellipse.	DMPs - limma (M_vals ~ Diagnosis + age + sex + blood cells (Houseman)).	Pvalue (non-adj) < 5e-8 and deltaM > 0.4.<br>Select CpGs using minimum-redundancy-maximum-relevance ensemble (mRMRe).<br>e1071 (SVM linear). 10fold cv.
Functional Insight into and Refinement of the Genomic Boundaries of the JARID2-Neurodevelopmental Disorder Episignature	2023/09	JARID2-syndrome	*JARID2*	IDAT – Minfi (Illumina normalization)<br>XY probes ruled out. PCA for outliers and batches.	DMPs - limma (M_vals ~ Diagnosis + blood cells (Houseman)).<br>DMRs – DMRcate	deltaBeta > 0.05. Order and select top 1000 (-log(Pval) * deltaMethy) and top 250 AUC and R^2.<br>e1071 (SVM linear). Repeated 4 times.

Identification of a robust DNA methylation signature for Fanconi anemia	2023/11	Fanconi anemia	*FANCA* complementation group	IDAT – ChAMP and Minfi (Illumina normalization) XY probes ruled out. PCA for outliers and batches.	DMPs - limma (M_vals ~ Diagnosis + blood cells (Houseman)). DMRs – DMRcate	deltaBeta > 0.05. Order and select top 1000 (-log(Pval) * deltaMethy) and top 500 AUC and R^2. < 0.84. e1071 (SVM linear). 5fold CV repeated 4 times. SMOTE imbalance correction.
Epigenetics of Autism Spectrum Disorders: A Multi-level Analysis Combining Epi-signature, Age Acceleration, Epigenetic Drift and Rare Epivariations Using Public Datasets	2023/11	ASD		IDAT - RnBeads (SWAN normalization). DNAmage calculator for immune cell composition, epigenetic age and age acceleration.	DMPs and DMRs – using hierarchical linear models, corrected by age, batch effect, principal components (associate immune cell composition and group variable).	Pvalue (FDR and Bonferroni).
Rare de novo gain-of-function missense variants in DOT1L are associated with developmental delay and congenital anomalies	2023/11	DOT1L associated syndrome	*DOT1L*	IDAT – Minfi (Illumina normalization). Probes with beta 0 or 1 in >0.25 samples. XY probes ruled out.	DMPs – limma(Beta_vals ~ Diagnosis + Age + PC1).	P. value (BH) < 0.3 & deltaBeta > 0.1.
Comprehensive evaluation of the implementation of episignatures for diagnosis of neurodevelopmental disorders (NDDs)	2023/12	Kabuki	*KDM2D*	IDAT – Minfi (Raw, illumina, Swan, Quantile, Noob and Funnorm normalization) XY probes ruled out.	DMR – bumphunter in ChAMP	Scikit-learn SVM, Random Forest and Penalized Logistic Regression
		Sotos	*NSD1*	IDAT – Minfi (Raw, illumina, Swan, Quantile, Noob and Funnorm normalization) XY probes ruled out.	DMR – bumphunter in ChAMP	Scikit-learn SVM, Random Forest and Penalized Logistic Regression
Clinico-biological refinement of BCL11B-related disorder and identification of an	2024/01	BCL11B-related disorder (IDDSFTA)	*BCL11B*	IDAT – Minfi (Illumina normalization) XY probes ruled out. PCA for outliers and batches.	DMPs - limma (M_vals ~ Diagnosis + blood cells (Houseman)). DMRs – DMRcate	deltaBeta > 0.05. Order and select top 800 (-log(Pval) * deltaMethy) and top 200 AUC and R^2 < 0.9.

episignature: A series of 20 unreported individuals						e1071 (SVM linear). Separate cohort in 75% training and 25% testing.
Identification of the DNA methylation signature of Mowat-Wilson syndrome	2024/02	Mowat-Wilson	*ZEB2*	IDAT – Minfi (Illumina normalization)<br>XY probes ruled out. PCA for outliers and batches.	DMPs - limma (Beta_vals ~ Diagnosis + blood cells (Houseman)).	No specified filters<br><br>e1071 (SVM linear). Separate cohort in 75% training and 25% testing. 20 iterations 25-leave-out CV.
A new blood DNA methylation signature for Koolen-de Vries syndrome: Classification of missense KANSL1 variants and comparison to fibroblast cells	2024/03	Koolen-de Vries	*KANSL1*, 17q21.31 del	IDAT – Minfi (Illumina normalization)<br>XY probes ruled out. PCA for outliers and batches.	DMPs - limma (Beta_vals ~ Diagnosis + age + sex blood cells (Houseman)).	Pvalue (FDR) < 0.05 and deltaBeta > 0.1.<br><br>Caret (SVM).
DNA methylation episignature, extension of the clinical features, and comparative epigenomic profiling of Hao-Fountain syndrome caused by variants in USP7	2024/03	Hao-Fountain	*USP7*	IDAT – Minfi (Illumina normalization)<br>XY probes ruled out. PCA for outliers and batches.	DMPs - limma (M_vals ~ Diagnosis + blood cells (Houseman)).<br>DMRs – DMRcate	deltaBeta > 0.05. Order and select top 800 (-log(Pval) * deltaMethy) and top 400 AUC and $R^2 < 0.8$.<br><br>e1071 (SVM linear). Separate cohort in 75% training and 25% testing.
Identification of DNA methylation episignature for the intellectual developmental disorder, autosomal dominant 21 syndrome, caused by variants in the CTCF gene	2024/03	Intellectual developmental disorder, autosomal dominant 21	*CTCF*	IDAT – Minfi (Illumina normalization). XY probes ruled out. PCA for outliers and batcher	DMPs – limma (M_vals ~ Diagnosis + blood cells (Housemann)).<br>DMRs – DMRcate	Order and select top 800 (-log(Pval) * deltaMethy) and top 400 AUC and $R^2 < 0.9$<br><br>e1071 (SVM linear). Separate cohort in 75% training and 25% testing.

Loss of function in NSD2 causes DNA methylation signature similar to that in Wolf-Hirschhorn syndrome	2024/03	Wolf-Hirschhorn syndrome	*NSD2*, 4q16.13	No specified.	DMPs – limma (M_vals ~ Diagnosis + blood cells + gender + batch (Housemann)).	deltaBeta > 0.2 & P.value < 2.4e-16 (bonferroni).<br><br>SVM. Leave-one-out CV.
EHMT1 pathogenic variants and 9q34.3 microdeletions share altered DNA methylation patterns in patients with Kleefstra syndrome	2024/06	9q34.3 microduplicatio n syndrome	9q34.3	IDAT – Minfi (Illumina normalization). XY probes ruled out. PCA for outliers and batcher	DMPs – limma (M_vals ~ Diagnosis + blood cells (Housemann)).<br>DMRs – DMRcate	Order and select top 800 (-log(Pval) * deltaMethy) and top 400 AUC and $R^2 < 0.7$<br><br>caret (SVM linear). Separate cohort in 75% training and 25% testing.
DNA methylation episignature and comparative epigenomic profiling for Pitt-Hopkins syndrome caused by TCF4 variants	2024/07	Pitt-Hopkins	*TCF4*	IDAT – Minfi (Illumina normalization)<br>XY probes ruled out. PCA for outliers and batches.	DMPs - limma (M_vals ~ Diagnosis + blood cells (Houseman)).<br><br>DMRs – DMRcate	No filters specified.<br><br>e1071 (SVM linear). Separate cohort in 75% training and 25% testing.
The detection of a strong episignature for Chung–Jansen syndrome, partially overlapping with Börjeson–Forssman–Lehmann and White–Kernohan syndromes	2024/07	Chung–Jansen	*PHIP*	IDAT – Minfi (Illumina normalization)<br>XY probes ruled out. PCA for outliers and batches.	DMPs - limma (M_vals ~ Diagnosis + blood cells (Houseman)).<br><br>DMRs – DMRcate	deltaBeta > 0.05. Order and select top 800 (-log(Pval) * deltaMethy) and top 500 AUC and $R^2 < 0.75$.<br><br>e1071 (SVM linear). Separate cohort in 75% training and 25% testing.
MSL2 variants lead to a neurodevelopmental syndrome with lack of coordination, epilepsy, specific dysmorphisms, and a distinct episignature	2024/07	MSL2-related NDDs	*MSL2*	IDAT – Minfi (Illumina normalization)<br>XY probes ruled out. PCA for outliers and batches.	DMPs - limma (M_vals ~ Diagnosis + blood cells (Houseman)).<br><br>DMRs – DMRcate	deltaBeta > 0.05. Order and select top 900 (-log(Pval) * deltaMethy) and top 450 AUC and $R^2 < 0.75$.

						e1071 (SVM linear). Separate cohort in 75% training and 25% testing. 10fold CV.
CUL3-related neurodevelopmental disorder: Clinical phenotype of 20 new individuals and identification of a potential phenotype-associated episignature	2024/07	NEDAUS	*CUL3*	IDAT – Minfi (Illumina normalization)<br>XY probes ruled out. PCA for outliers and batches.	DMPs - limma (M_vals ~ Diagnosis + blood cells (Houseman)).<br><br>DMRs – DMRcate	deltaBeta > 0.05. Order and select top (-log(Pval) * deltaMethy) and top AUC and R^2.<br><br>e1071 (SVM linear). Separate cohort in 75% training and 25% testing. 10fold CV.
Menke-Hennekam syndrome; delineation of domain-specific subtypes with distinct clinical and DNA methylation profiles	2024/07	Menke-Hennekam	*CREBBP*, *EP300*	IDAT – Minfi (Illumina normalization)<br>XY probes ruled out. PCA for outliers and batches.	DMPs - limma (M_vals ~ Diagnosis + blood cells (Houseman)).<br><br>DMRs – DMRcate	3 models: ZZ, TAZ2 and ID4.<br><br>deltaBeta > 0.05. Order and select top 1000/600/900 (-log(Pval) * deltaMethy) and top 333/300/225 AUC and R^2. < 0.85/0.8/0.75 (respectively)<br><br>e107 (SVM linear). Separate cohort in 75% training and 25% testing. 10fold CV.
Diagnostic utility of DNA methylation analysis in genetically unsolved pediatric epilepsies and CHD2 episignature refinement	2024/08	DEEs, Epilepsy	*CHD2*	IDAT – Minfi. PCA for outliers (only betas in chr1). ComBat for batch correction	DMPs – limma (M_vals ~ Diagnosis + blood cell (Houseman)).<br><br>Epimutation – 1kb sliding window, 3 CpGs must be outliers (beta value) based on	Order and select top 1000 (-log(Pval) * deltaMethy, top 200 AUC and $R^2 < 0.85$.<br>No ML model.

					99.25th percentile + 0.15 / 0.75th percentile – 0.15.	
Identification of a DNA methylation episignature for recurrent constellations of embryonic malformations	2024/08	Recurrent constellations of embryonic malformations (RCEM)		IDAT – Minfi (Illumina normalization) XY probes ruled out. PCA for outliers and batches.	DMPs - limma (M_vals ~ Diagnosis + blood cells (Houseman)). DMRs – DMRcate	Identified a robust and an intermediate group. deltaBeta > 0.05. Order and select top 900/800 (-log(Pval) * deltaMethy) and top 450/400 AUC and R^2. < 0.65/0.95 (respectively) e107 (SVM linear). Separate cohort in 75% training and 25% testing. Leave-one-out CV.
Pathogenic variants in KMT2C result in a neurodevelopmental disorder distinct from Kleefstra and Kabuki syndromes	2024/08	KMT2C-related syndrome	*KMT2C*	IDAT – Minfi XY probes ruled out. Eliminate samples putative of falling in SNVs sites (MethylToSNP R package)	DMPs - limma (M_vals ~ Diagnosis + monocytes + batch + PC1 + PC2 (Houseman)).	pval FDR < 0.05, deltaBeta > 0.1 caret (SVM linear).). Separate cohort in 75% training and 25% testing. Leave-one-out CV.
Genome-wide epigenetic signatures facilitated the variant classification of the PURA gene and uncovered the pathomechanism of PURA-related neurodevelopmental disorders	2024/08	PURA-related neurodevelopm ental disorders	*PURA*, 5q31.3 deletion	IDAT – Minfi (BMIQ). XY probes ruled out. PCA for batches. ComBat for batch correction.	DMPs – ChAMP DMRs - ChAMP	P.value (BH) < 0.05, deltaBeta > 0.1, AUC > 0.98. e1071 (SVM)

Microduplications of ARID1A and ARID1B cause a novel clinical and epigenetic distinct BAFopathy	2024/09	Non-syndromic duplications	*ARID1A, ARID1B*	IDAT – Minfi (Illumina normalization)<br>XY probes ruled out. PCA for outliers and batches.	DMPs - limma (M_vals ~ Diagnosis + blood cells (Houseman)).<br>DMRs – DMRcate	deltaBeta > 0.05. Order and select top (-log(Pval) * deltaMethy) and top AUC and R^2<br>e107 (SVM linear). Separate cohort in 75% training and 25% testing. Leave-one-out CV.
Epigenomic and phenotypic characterization of DEGCAGS syndrome	2024/10	DEGCAGS	*ZNF699*	IDAT – Minfi (Illumina normalization)<br>XY probes ruled out. PCA for outliers and batches.	DMPs - limma (M_vals ~ Diagnosis + blood cells (Houseman)).<br>DMRs – DMRcate	deltaBeta > 0.05. Order and select top 1000 (-log(Pval) * deltaMethy) and top 200 AUC and R^2<br>e107 (SVM linear). Separate cohort in 75% training and 25% testing. Leave-one-out CV.
Discovery of DNA methylation signature in the peripheral blood of individuals with history of antenatal exposure to valproic acid	2024/11	Fetal Valproate syndrome		IDAT – Minfi (Illumina normalization)<br>XY probes ruled out. PCA for outliers and batches.	DMPs - limma (M_vals ~ Diagnosis + blood cells (Houseman)).<br>DMRs – DMRcate	Decrease order (-log(Pval) * deltaMethy). Test best combination AUC and R^2 parameters with different n cases.<br>e107 (SVM linear). Separate cohort in 75% training and 25% testing. Leave-one-out CV.
Blepharophimosis with intellectual disability and Helsmoortel-Van Der Aa Syndrome share episignature and phenotype	2024/12	Blepharophimosis with intellectual disability and Helsmoortel-	*SMARCA2* (BIS), *ADNP* (HVDAS)	IDAT – Minfi (Illumina normalization)<br>XY probes ruled out. PCA for outliers and batches.	DMPs - limma (Beta_vals ~ Diagnosis + blood cells (Houseman)).<br>DMRs – DMRcate	deltaBeta > 0.05. Order and select top 1000 (-log(Pval) * deltaMethy) and top 200 AUC and R^2<br>e107 (SVM linear). Separate cohort in 75% training and

		Van Der Aa Syndrome				25% testing. Leave-one-out CV.
Discovery of a DNA methylation profile in individuals with Sifrim-Hitz-Weiss syndrome	2025/02	Sifrim-Hitz-Weiss syndrome	*CHD4*	IDAT – Minfi (Illumina normalization)<br>XY probes ruled out. PCA for outliers and batches.	DMPs - limma (M_vals ~ Diagnosis + blood cells (Houseman)).<br><br>DMRs – DMRcate	Order and select top 900 (-log(Pval) * deltaMethy) and 450 AUC and $R^2 > 0.8$.<br><br>e1071 (SVM non-specified kernel). Leave-one-out.
ARID2-related disorder: further delineation of the clinical phenotype of 27 novel individuals and description of an epigenetic signature	2025/03	ARID2-related disorder	*ARID2*	IDAT – Minfi (Illumina normalization)<br>XY probes ruled out. PCA for outliers and batches.	DMPs - limma (Beta_vals ~ Diagnosis + blood cells (Houseman)).<br><br>DMRs – DMRcate	Filters not specificed (P.value (BH), AUC and R^2).<br><br>e1071 (SVM non-specified kernel). 4fold CV.
Pleiotropic effects of MORC2 derive from its epigenetic signature	2025/04	Charcot-Marie-Tooth disease type2 Z (CMT2Z).<br>Spinal muscular atrophy (SMA).<br>Leigh syndrome (LS).<br>Mitochondrial disease (MD).<br>Cockayne syndrome (CS)	*MORC2*	IDAT – minfi (quantile normalization).<br>Common EPICv2 and EPICv1.<br>XY probes removed.	DMPs - limma (Beta_vals ~ Diagnosis + blood cells (Houseman)).<br><br>DMRs – DMRcate	Order and select top 1000 (-log(Pval) * deltaMethy) and AUC > 0.99.<br><br>e1071 (SVM non-specified kernel). 5-fold cv.
Dominant variants in major spliceosome U4 and U5 small nuclear RNA genes cause neurodevelopmental disorders through splicing disruption	2025/05	ReNU syndrome	*RNU4-2*	IDAT – meffil (functional normalization).<br>XY removed.	DMPs - meffil.ewas (~Mvals, blood cells (meffil), age (skinHorvath), gender).<br>5fold-cv based on pathogenicity	P.val < 10e-7, deltabeta > 0.05.<br>SVM (package no specified, nor the kernel) 5-fold CV.

Discovery of a DNA methylation episignature as a molecular biomarker for fetal alcohol syndrome	2025/09	Fetal Alcoholic Syndrome Disorder		IDAT – SeSAMe (pOOBAH approach inferred) XY probes removed. Common EPICv2 and EPICv1 probes.<br>PCA for batches	DMP limma (M_vals ~ Diagnosis + blood cells (Houseman)).<br><br>DMRs – DMRcate	Order and select top 900 (-log(Pval) * deltaMethy) and top 600 AUC and $R^2 > 0.6$<br>e1071 (SVM non-specified kernel). Separate cohort in 75% training and 25% testing. 4-fold CV
PTBP1 variants displaying altered nucleocytoplasmic distribution are responsible for a neurodevelopmental disorder with skeletal dysplasia	2025/09	PTBP1 associated syndrome	*PTBP1*	IDAT – SeSAMe (pOOBAH approach inferred) XY probes removed. Common EPICv2 and EPICv1 probes.<br>PCA for batches	DMP limma (M_vals ~ Diagnosis + blood cells (Houseman)).<br><br>DMRs – DMRcate	Order and select top X (not specified) (-log(Pval) * deltaMethy) and top X (not specified) AUC and $R^2 >$ X (not specified).<br>e1071 (SVM non-specified kernel). Separate cohort in 75% training and 25% testing. 4-fold CV
Characterization of snRNA-related neurodevelopmental disorders through the Spanish Undiagnosed Rare Disease Programs	2025/09	ReNU syndrome	*RNU4-2, RNU2-2*	IDAT – meffil (functional normalization).<br>XY removed.<br>ComBat for batch (sample pool)	DMP limma (M_vals ~ Diagnosis + blood cells (meffil) + age + sex).<br><br>DMRs – DMRcate	Order and select top 1000 (-log(Pval) * deltaMethy) and top 500 AUC and $R^2 > 0.6$.<br>No ML Model.
